# Enrichment, Source Apportionment and Health Risk Assessment of Soil Potentially Harmful Elements Associated with Different Land Use in Coastal Tidelands Reclamation Area, Eastern China

**DOI:** 10.3390/ijerph17082822

**Published:** 2020-04-19

**Authors:** Xinjian Chen, Sihua Huang, Xuefeng Xie, Ming Zhu, Jianguo Li, Xiaohan Wang, Lijie Pu

**Affiliations:** 1 School of Geography and Ocean Science, Nanjing University, Nanjing 210023, China; dg1327009@smail.nju.edu.cn (X.C.); huangsihua@smail.nju.edu.cn (S.H.); zhumingnju@126.com (M.Z.); 2 The Key Laboratory of the Coastal Zone Exploitation and Protection, Ministry of Natural Resources, Nanjing 210023, China; 3 College of Geography and Environmental Sciences, Zhejiang Normal University, Jinhua 321004, China; 4 School of Geography, Geomatics and Planning, Jiangsu Normal University, Xuzhou 221116, China; lijianguo531@126.com; 5 Institute of Environment and Sustainable Development in Agriculture, Chinese Academy of Agricultural Sciences, Beijing 100081, China; wangxiaohan0203@163.com

**Keywords:** potentially harmful elements enrichment, tideland reclamation, land use, health risk, eastern China

## Abstract

Coastal tidelands are important ecological habitat resources and valuable resources for agricultural land reclamation. Enrichment of potentially harmful elements (PHEs) in soil caused by anthropogenic activity is an important factor implicated in the ecological deterioration of soil in China. A total of 54 soil sample sites were selected from a 30-year reclaimed tideland and an adjoining coastal wetland. Descriptive and multivariate statistical analyses were performed to describe the enrichment, source, health risk status of eight PHEs (As, Co, Cr, Cu, Mn, Ni, Pb, and Zn) after long-term reclamation. Results indicated that after 30 years of reclamation, most soil PHEs are slightly enriched, whereas no serious threat of environmental pollution was observed. In the reclamation area, the enrichment of PHEs in the aquaculture land, industrial land, and cropland was relatively high compared with other land use types, such as tideland and halophyte land. The source analysis divided the PHEs into five categories: (1) Cu; (2) Co and Mn; (3) Cr; (4) As and Pb; (5) Zn and Ni. Cu was completely derived from natural parent materials and other elements were governed by both weathering of parent rock and human activities, including agricultural activities, industrial production, and transportation emissions. The health risk assessment showed that the soil PHEs potentially had no non-carcinogenic risk to the public, but there was an acceptable probability to have cancer due to Cr and As. Meanwhile, children are more susceptible to harm from the PHEs in soil than adults. According to the economic and social development situation in the coastal region, it is necessary to pay attention to the environmental threats of PHEs enrichment.

## 1. Introduction

The enrichment of potentially harmful elements (PHEs) in soil has been widely concerned as a significant ecological and environmental issue [1]. The bedrock geochemistry and human activities are the main sources of PHEs in the environment [2,3]. Lee et al. [4] reported that natural soils and rocks contain a high content of PHEs, such as Cd and Mo. Usually, the enrichment of soil PHEs are mainly caused by anthropogenic factors (industrialization, urbanization, agriculture, and transportation), and the enrichment of PHEs tend to aggravate with the increase of economic development and human activities [3,5]. The accumulation of PHEs appeared in a typical rapid urbanization city of China, specifically, the concentration of PHEs gradually decreased with the increase of distance from the constructed land, which indicated the significant effect of the urbanization process [6]. The concentration of soil PHEs is also affected by land-use and rises along with a corresponding increase in land-use intensity [3,7]. Li et al. [8] assessed the human health risk caused by soil PHEs pollution from mines in China and indicated that Cd, Pb, Cu, Zn, Hg, As, and Ni as the priority control elements. The high PHEs concentration in the industrial zone is mainly derived from the discharge of poorly treated “three wastes” (waste gas, water, and residues), which enters the soil through leaching, settlement, and run-off during rains [9]. It is reported that steel smelting is an important source to elevate Cd, Pb, and Zn [10]. The fossil fuel combustion from a coal-fired plant will cause the enrichment of Pb [10], while the concentration of Cd in the soil around the ferroalloy plant is significantly higher [11]. Zhang et al. [12] showed that the total pollution rate of farmland soil in China was 10.18%, mainly from Cd, Hg, Cu, and Ni. The external sources of farmland were agricultural activities (e.g., animal manure, inorganic fertilizers, and pesticides) in remote areas and industrial sewage irrigation in the suburbs [12]. Besides, Zn, Cd, Pb, Cr, Ni, and Cu are relatively high in the infrastructure area of the transportation (e.g., highway, railway) and its surrounding areas, which may originate from fuel combustion in vehicles and abrasion of tires and roads/tracks [8,13]. In addition, land-use alters the soil texture, chemical composition, and microbial activity, thus leading to corresponding changes in the uptake and transfer capacity of soil PHEs [7,14,15].

The material flows and energy cycles between ecosystems have infinitely amplified the threat of soil PHEs to the survival and development of animals and humans [5]. Due to its non-biodegradability and persistence, PHEs damage the physicochemical properties of soil after long-term accumulation, leading to nutrient loss and degradation [16]. Meanwhile, soil PHEs will further threaten human health through the food chain with the pathways of ingestion, inhalation and dermal contact [3]. For example, Cd, Pb, and As are proven to be closely related to cancer, kidney dysfunction, nervous system dysfunction, and skin damage [1,3,16].

The coastal zone is an intersection between land and ocean where 25% of global primary productivity occurs, and it supplies nearly 70% of global fish catch [17,18]. The coastal zone only occupies 12% of the Earth’s surface, while supports about 50% of the world’s population [18]. In many coastal countries and regions, sea reclamation (e.g., Netherlands, South Korea, and Japan), and tidal land reclamation (e.g., China, Vietnam) are the dominant ways to accommodate the increasing demand for space for living and development [19,20]. Anthropogenic activities such as marine industry, tourism, fisheries, and shipping have extremely promoted the economic development of the coastal zone, however, they have also brought a series of ecological and environmental crises, including the decline of ecosystem services, destruction of biodiversity, metal and nutrient pollution [17,18,21]. It is widely reported that coastal fishery farming, agricultural cultivation, energy (wind, solar, etc.) base construction, industrial construction, and port construction lead to changes in the soil’s PHEs concentration and the potential ecological threats [21,22,23]. Currently, a series of studies have been carried out on PHEs in the coastal zone, including the spatial pattern and source analysis [1], pollution assessment [24], ecological risk assessment [25], the influences of land use on distribution and morphology of PHEs [14,26]. However, few studies have focused on the health risks of PHEs associated with different land use in the coastal tidelands reclamation area [22]. The coastal zone of Jiangsu Province is one of the most economically developed areas of China where the reclaimed tidal flats were up to 3350 km^2^ from 1950 to 2015, which provided important reserve land resources for regional urbanization and industrial development. In 2009, the State Council of China implemented a national strategy “*development planning in Jiangsu coastal areas*”, which plans to reclaim 1805 km^2^ coastal tidal flats in Jiangsu from 2009 to 2020. The high-intensity, rapid reclamation of coastal zones within a short time period has affected the physicochemical properties of soil in tidelands and even accelerated the rapid enrichment of pollutants [14,27]. There are over ten ports along the coast of Jiangsu Province, which undertake the transportation of numerous import and export commodities and energy (coal, petroleum and metal ore, etc.). Furthermore, port-centered industries such as metallurgy and petrochemicals, electricity, cement, and papermaking have also developed rapidly, which have posed huge threats to the soil ecosystem. Hence, the investigation of characteristics and trends of PHEs enrichment in the soil after coastal reclamation is important to clarify the influence of anthropogenic activities on tidelands and to understand and assess the ecological and environmental effects of coastal reclamation.

In this study, multivariate statistical methods and a US EPA soil health risk assessment model were applied to explore the enrichment, source, and health risk of PHEs in a long-term reclaimed coastal area. The objectives of this study are (1) to explore the variation of soil PHEs under different land use; (2) to identify the source of soil PHEs in a coastal reclamation area; and (3) to assess the level of health risk status after long-term reclamation. Our study is designated to provide a scientific basis for the monitoring and pollution control of the tideland.

## 2. Materials and Methods

### 2.1. Study Area

**Figure 1:**
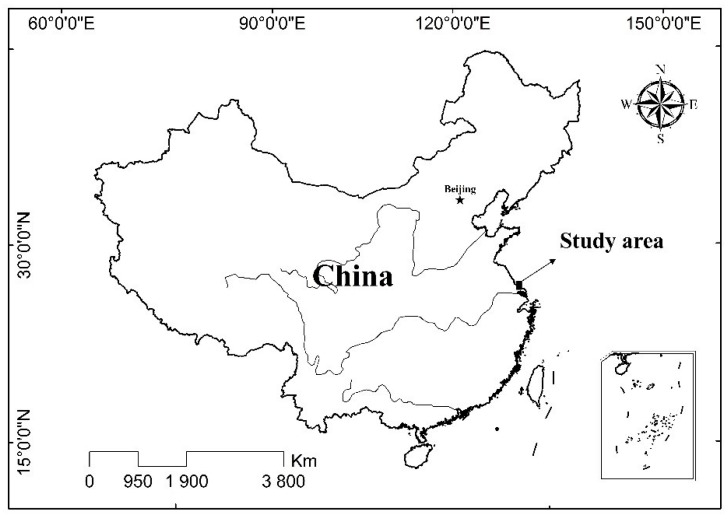
The location of the study area.

The study area is located in the coast of the Dongtai City (120°78′86.7″–120°90′29.2″ E; 32°95′22.3″–33°00′38.9″ N) with an area of 200 km^2^, which closely adjoins Yancheng National Nature Reserve, Jiangsu Province (Figure 1). The regional climate is characterized as a northern humid subtropical monsoon climate with four distinct seasons, and the annual average rainfall and temperature are 1035 mm and 15.0 °C, respectively. The soils in the study area are mainly derived from the modern marine and fluvial sediments, which belong to sandy loam and are classified as coastal saline-sodic soils. Actually, the study area is considered one of the fastest silting regions in eastern China, and the tidal land is currently expanding seaward at a rate of 200 m per year [20]. The reclamation of the study area mainly depended on different land uses to carry out the process of desalination after the construction of seawalls. The land-use/cover types varied with increasing reclamation years and occurred in three stages [28]. In the early period of reclamation, newly reclaimed tidal land changed to aquaculture ponds and salt-tolerant crops, such as *Sesbania cannabina*. Over 10–30 years, the land-use pattern gradually turned into cropland with an increase of soil fertility and decrease of soil salinity, and some crops were planted in this period, such as cotton, wheat, and corn. After 30 years of reclamation, soil quality basically met the cultivation for most crops with consistent cultivation and fertilization, such as paddy, wheat, corn, rapeseed, and broad bean. In addition, driven by the demands of economic development, some reclaimed land has been converted into industrial land to support industries such as power generation, textiles, chemicals, and automobile infrastructure.

### 2.2. Sampling Design and Chemical Analysis

**Figure 2:**
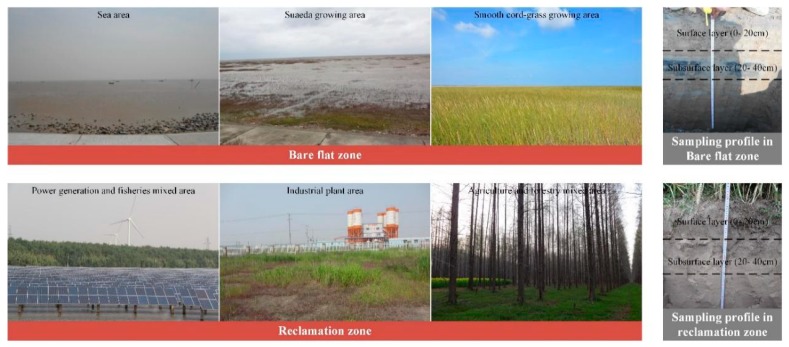
Typical land use and sample profile in the study area.

Soil sites were chosen in mid-August 2014 by using a uniform grid method in the study area. In total, 54 soil sampling sites were collected, including 5 sites respectively in tideland and halophyte land in coastal wetlands, and 44 sites (industrial land (10), forestland (6), residential land (7), aquaculture land (6), cropland (8), and vegetable land (7)) in the areas reclaimed for 30 years. Both surface (0–20 cm) and subsurface (20–40 cm) samples were collected from each sampling site and recorded using GPS (Figure 2). Each soil sample consisted of 4–8 subsamples collected in a surrounding 10-m area. After air-drying at room temperature, the soil samples were grounded and sieved through a 2 mm nylon sieve to remove animal and plant residues, as well as other impurities, and then sieved through a 0.149 mm nylon sieve as described by Xie et al. [29]. From the sieved soil, 10–15 g preliminarily treated soil samples were used to form soil tablets. Eight PHEs (As, Co, Cr, Cu, Mn, Ni, Pb, and Zn) were analyzed according to X-ray fluorescence spectrometry methods, as described by Xia et al. [30].

### 2.3. Descriptive and Multivariate Statistical Analysis

Descriptive analysis (range, mean, standard deviation, coefficient of variation) was applied to understand the distribution and variation of PHEs in the study area. The differences of PHEs among different land-use types were compared using the analysis of variance (ANOVA) procedure and the mean comparisons were performed using the Fisher’s least significant difference (LSD) test with a probability defined at 0.05 [24]. Pearson’s correlation analysis and cluster analysis (CA) are effective to identify the source of PHEs in soil [15]. Pearson’s correlation analysis could determine whether the sources of PHEs are the same through the correlation between PHEs, and CA could provide variable grouping results, which corroborates their correctness with correlation analysis results. Descriptive and multivariate statistical analysis was conducted in SPSS 22.0 for Windows (SPSS München, Germany).

The enrichment factor (EF) is applied to determine the degree of PHEs enrichment and to differentiate between elements originating from human activities and those from natural provenance by using Equation (1) [1,31].

(1) $$EF=(\frac{M_{x}}{N_{ref}}{)}_{sample}\times /(\frac{M_{x}}{N_{ref}}{)}_{background}$$

where Mx and *Nref* are the concentration of PHEs and reference elements in soil, respectively. In this study, Fe was adopted as a reference because it is one of the abundant elements of soil [31]. The geochemical baseline of Fe (4.5 μg/g) in Jiangsu Province was chosen as reference background values [32]. Generally, EF is applied to differentiate whether the element originates from anthropogenic activities. The element is completely derived from natural processes under EF < 1, while it might be from man-made sources under EF > 1 and the proportion of human sources increased with the increasing of EF [33]. The EF is also applied to estimate polluted degree, which can be divided into smaller enrichment (EF < 2), moderate enrichment (2 ≤ EF < 5), significant enrichment (5 ≤ EF < 20), highly enrichment (20 ≤ EF < 40) and extremely high enrichment (EF ≥ 40) [31,33].

### 2.4. Health Risk Assessment

The long-term accumulation of PHEs in soil may cause a threat to the health of animals and human beings, and increased risk of cancer [31,34]. The methodology used in this study to assess the exposure risks of PHEs to human health in the coastal reclaimed area based on risk assessment guidance for superfund (RAGS) health risk assessment as developed by the United States Environmental Protection Agency (US EPA) [35]. Direct exposure of an individual to PHEs in the soil through three main pathways: ingestion, inhalation, and dermal contact [36,37]. The average daily dose through three pathways can be respectively estimated by the following Equation [8].

(2) $$ADD_{ing}=C_{soil}\times \frac{IngR\times CF\times EF\times ED}{BW\times AT_{nc/ca}}$$

(3) $$ADD_{inh}=C_{soil}\times \frac{InhR\times EF\times ED}{PEF\times BW\times AT_{nc/ca}}$$

(4) $$ADD_{derm}=C_{soil}\times \frac{SA\times CF\times SL\times ABS\times EF\times ED}{BW\times AT_{nc/ca}}$$

Where *ADD_ing_*, *ADD_inh,_* and *ADD_derm_* are the average daily dose from ingestion, inhalation, and dermal contact, respectively (mg·kg^−1^·d^−1^). *C_soil_* is the concentration of PHEs in soil (mg·kg^−1^·d^−1^). Related definition and parameters used in the average daily dose of soil metals can be found in Table 1.

**Non-carcinogenic risk assessment:** Hazard quotient (HQ) is calculated by the ratio of average daily dose to the corresponding reference dose, which is used to assess the potential non-carcinogenic risk of PHEs under certain exposure pathways. The Hazard Index (HI) is the sum of the HQ and represents the overall potential non-carcinogenic effects posed by all exposure pathways. The HQ of a single element is determined by Equation (5) [35].

(5) $$HI=\sum_{i=1}^{n}\sum_{j=1}^{m}HQ_{ij}=\sum_{i=1}^{n}\sum_{j=1}^{m}\frac{ADD_{ij}}{RfD_{ij}}$$

(6) $$CR=\sum_{i=1}^{n}\sum_{j=1}^{m}CR_{ij}=\sum_{i=1}^{n}\sum_{j=1}^{m}SF_{ij}\times ADD_{ij}$$

where *n* =8, *i* = As, Co, Cr, Cu, Mn, Ni, Pb, and Zn, *m* =3, *j* = ingestion, inhalation and dermal contact. *RfD_ij_* is the reference dose of *i* element in *j* exposure pathway (mg·kg^−1^·d^−1^).

**Carcinogenic risk assessment:** Carcinogenic risk (CR) is the probability of cancer, which can be calculated by the product of daily average exposure multiplied by the corresponding slope factor. The CR of a single element is determined by Equation (6) [35]. *SF_ij_* is the slope factor *i* metal in *j* exposure pathway (mg·kg^−1^·d^−1^). The values of *RfD_ij_* and *SF_ij_* are shown in Table 2 [41,42,43].

If *HQ* or *HI* < 1, the non-carcinogenic risk did not exist to the exposed individual. However, if *HQ* or *HI* ≥ 1, the non-carcinogenic effect may occur with a probability which tends to increase with the increase of *HQ* or *HI* [8]. If *CR* < 10^−6^, it will neglect the carcinogenic risk to human health. 10^−6^ < *CR* < 10^−4^ is considered an acceptable range and *CR* > 10^−4^ is viewed as unacceptable [44].

## 3. Results

### 3.1. Descriptive Analysis

The average concentrations of soil PHEs (0–40 cm) showed that As, Co, Cr, Cu, Mn, Ni, Pb, and Zn were 3.31, 8.00, 52.50, 8.10, 458.76, 21.47, 15.90, and 45.45 mg·kg^−1^, respectively (Table 3). Compared with the Chinese Soil Environmental Quality Standard (*GB 15618-1995*), the average concentrations of PHEs were all within the range. In general, the concentrations of PHEs in the tidal flat reclamation area were relatively low, which is similar to the conclusions of other researches in Jiangsu coastal areas, and might be related to the local geochemical background [45,46]. Lv et al. [47] demonstrated that the background concentrations of Cr, Cu, Ni, Pb, and Zn in marine sediments were significantly lower than that in river alluvial deposits, lagoonal facie sediments, and delta sediments. However, the highest concentrations of Pb and Cr exceeded the limit values of the first and second-grade environmental quality standard for soils, respectively. This showed that partial land use had the opportunity to cause soil pollution, which might pose a threat to the regional natural background or agricultural production. Specifically, the average values of eight elements (As, Co, Cr, Cu, Mn, Ni, Pb, and Zn) did not exceed regional soil background values and the maximum values were close to or exceeded that value. The variation coefficient identified that As has a high coefficient of variation (60.06%), which suggests that the distribution of As is extremely non-uniform. The distribution of Cr, Cu, and Pb have been relatively moderate and non-uniform, owing to their coefficients of variation ranging from 20–30%. Although the average values of PHEs were lower than the risk control standard and regional soil background values, the human health risk of PHEs needs to be of concern due to the strong variation of PHEs (As, Cr, Cu, and Pb) and the difference between the exposure parameters and chronic reference dose of human health risk.

### 3.2. PHEs Concentrations under Different Land Use

**Figure 3:**
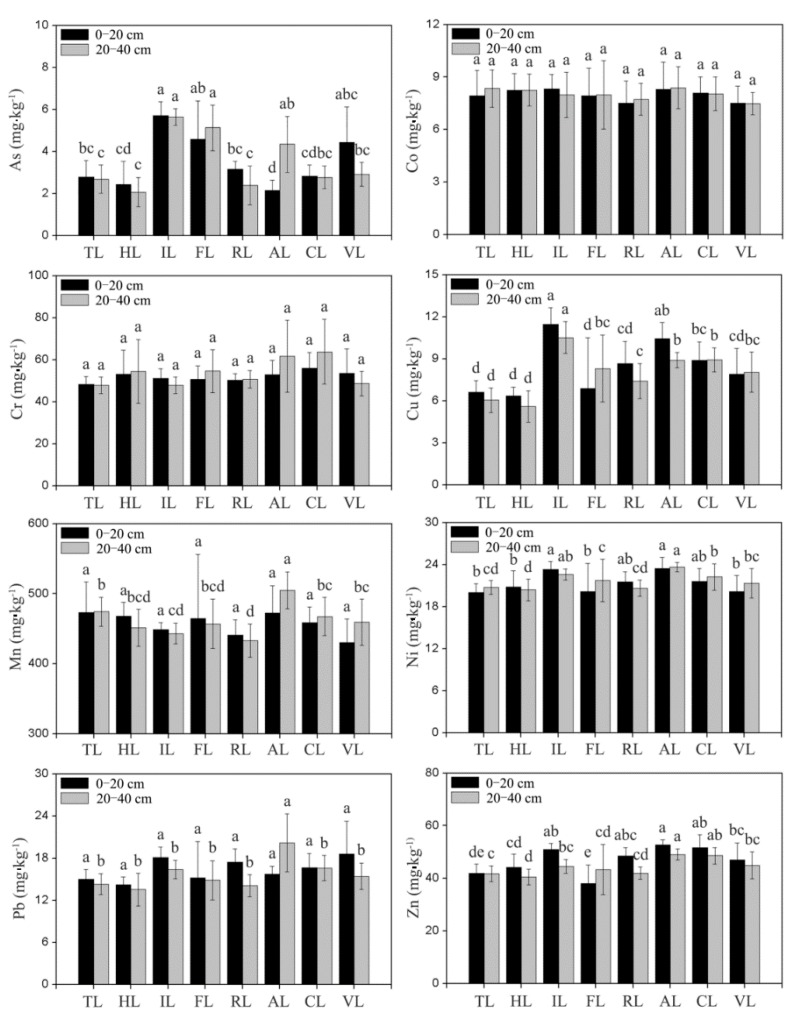
Enrichment of soil PHEs under different land use (TL: Tideland; HL: Halophyte land; IL: Industrial land; FL: Forestland; RL: Residential land; AL: Aquaculture land; CL: Cropland; VL: Vegetable land; Values are means of three replicates ± S.D; error bars refer to standard deviation; values having different lowercase letters indicated differences significantly (LSD, *p* < 0.05).

As shown in Figure 3, the average concentrations of As, Cu, Mn, Ni and Zn in surface and subsurface soil were observed to be significantly different from different land-use types (*p* < 0.05), which indicates that PHEs are greatly affected by the land use. However, soil depth had less effect on the concentrations of determined elements. The concentrations of Co and Cr did not change significantly under different land-use types, whatsoever, in surface or subsurface soil. However, the average concentration of Pb changed significantly in subsurface soil with the highest concentration observed in aquaculture land, and the concentration of As varied significantly under different land-use types with the highest concentration observed in industrial land. The average concentration of Cu in industrial land and aquaculture land was obviously higher than that of tideland and halophyte land, and the differences of other land-use types were not significant. Besides, no significant differences of Mn were observed in surface soil under different land-use patterns, whereas in subsurface soil, it was significantly higher in aquaculture land than that of other land-use types. Compared with the industrial land and aquaculture land, the concentrations of Ni and Zn in tideland, halophyte land, forestland, and vegetable land were relatively lower and changed mildly. The marine sediment sources and the degree of disturbance of human activities revealed that the lower concentration of PHEs was distributed in uncultivated soil (Tideland and Halophyte land) [49,50]. As, Cu, Pb, and Zn of industrial land, residential land, aquaculture land, cropland, and vegetable land were relatively higher than that of forestland, which might be caused by the absorption of trees and the re-entry of toxic elements from industrial activities and agricultural practices [14].

### 3.3. Source Analysis of PHEs in Soil

**Figure 4:**
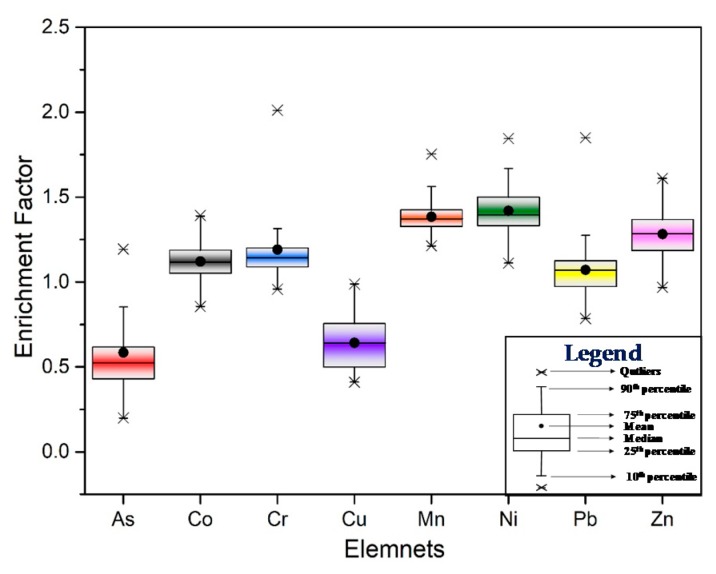
Boxplot of enrichment factors (*EFs*) for PHEs in the study area.

**Figure 5:**
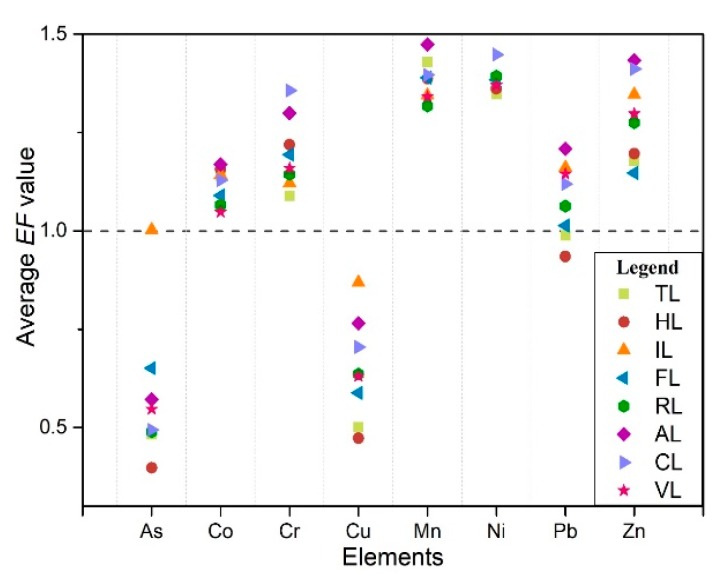
Scatter of average enrichment factors (*EFs*) for PHEs under different land-use types.

As presented in Figure 4, the *EF* of measured elements were in the following order: Ni > Mn > Zn > Cr > Co > Pb > Cu > As. The average *EF* of measured elements ranged from 1.0 to 1.5, except for Cu and As, which indicated that Co, Cr, Mn, Ni, Pb, and Zn were at minimal enrichment level. However, the highest *EF* of Cr, Mn, Ni, Pb, and Zn were above 1.5 and that of Cr was above 2.0, which was close to the medium enrichment level. This might be affected by anthropogenic activities (e.g., land use). As shown in Figure 5, the halophyte land, tideland, and forestland belong to the low *EF* value group whereas the cropland and aquaculture land belong to the high EF value group. It was worth noting that the average value of Cu was less than 1.0 in any kind of land-use types, which indicates that it was mainly controlled by the parent material in the study area. Only in industrial land did the *EF* value of As exceed 1.0, indicating that the As might be mainly affected by the parent material and local industrial activities such as steel smelting might be another reason [48]. For Pb, the mean EF of halophyte land, tideland and forestland were less than 1.0, while that of other land-use types was more than 1.0, especially in aquaculture land and industrial land. Similarly, the mean *EF* of Co ranged between 1.0–1.2 and was relatively high in aquaculture land and industrial land. Therefore, there was a slight enrichment of Pb and Co in the study area, which might be partly due to the natural processes, and local aquaculture and industrial activities. In addition, the average *EF* of Cr, Mn, Ni and Zn under different land-use types were more than 1.0, and the cropland and aquaculture land was much higher than that of other land use types, indicating that they were mainly controlled by agricultural and aquaculture activities.

**Figure 6:**
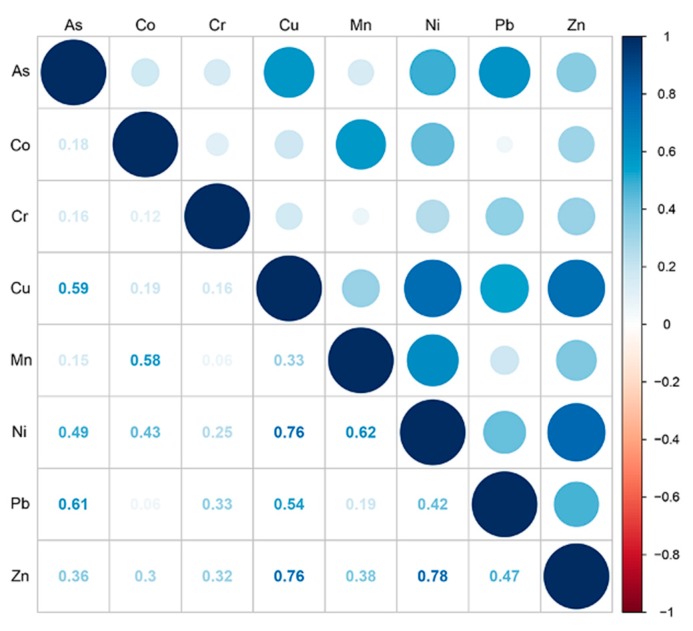
The correlation analysis of PHEs in 0–40 cm soil layer.

**Figure 7:**
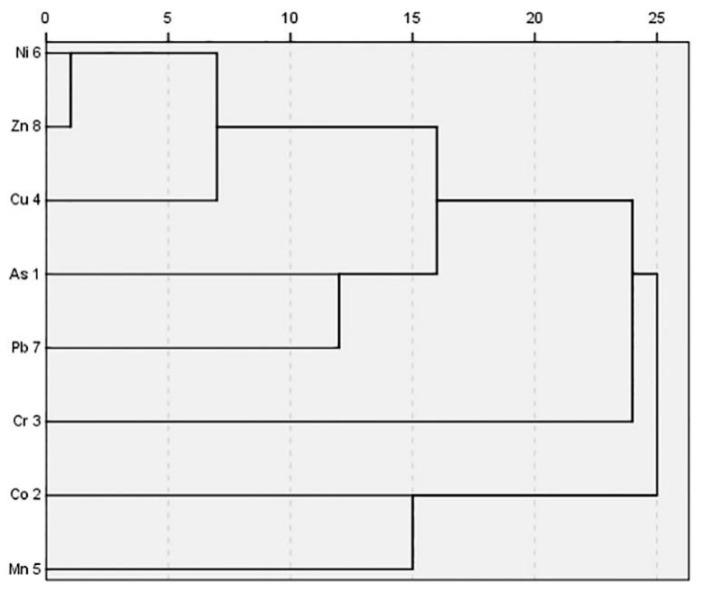
Dendrogram of PHEs concentrations in 0–40 cm soil layer.

Correlation analysis was performed to analyze the relationship between PHEs [36]. As depicted in Figure 6, Cr was weakly or uncorrelated with other elements (r < 0.4), which demonstrated the source of Cr was different from other measured elements. Most of the remaining PHEs were significantly correlated (r > 0.5). Especially, As-Cu-Pb had significant correlations (r > 0.5), Mn had significant correlation with Ni, Co (r = 0.62, 0.58), and Ni-Zn-Cu had extremely significant correlations (r > 0.75). As, Cu, and Pb had homology; part of the source of Mn might be the same as Ni and the other part might be the same as Co; Ni, Zn, and Cu had homology. The results of cluster analysis (Figure 7) indicated that all soil PHEs after reclamation were divided into four categories: (1) Co and Mn; (2) Cr; (3) As and Pb; (4) Cu, Zn and Ni. This was consistent with the results of the correlation analysis, indicating that PHEs in the study area were multi-sourced.

### 3.4. Health Risk Assessment

#### 3.4.1. Non-Carcinogenic Risk Assessment from Eight PHEs

**Figure 8:**
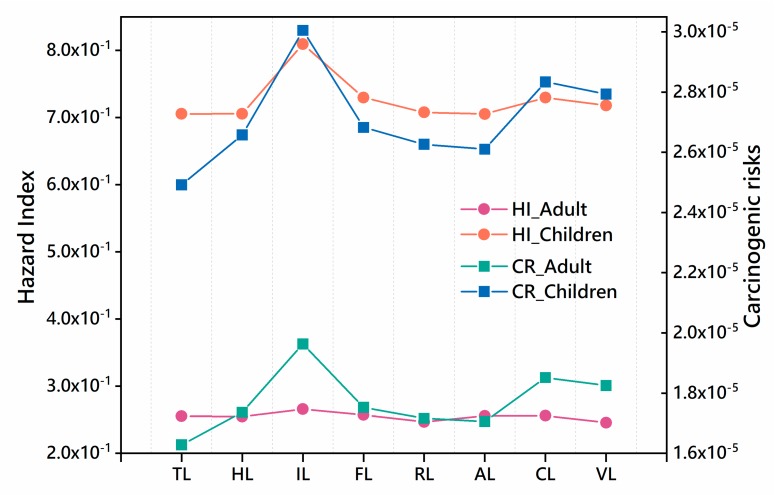
Carcinogenic risk index and non-carcinogenic risk index for adults and children under different land-use types.

According to the risk assessment model, the non-carcinogenic risk exposure dose (*ADD*), hazard quotient (*HQ_i_*), and hazard index (*HI*) of PHEs for adults and children through three exposure pathways (ingestion, inhalation, and dermal contact) were calculated. It could be seen from Table 4 that both the *HQ_i_* and the *HI* were less than 1, indicating that the PHEs in soil potentially had no non-carcinogenic risk to the public. Compared with the national average of non-carcinogenic risks, the non-carcinogenic risks of soil Cr, Cu, and Ni in the study area were lower than the national average, while the non-carcinogenic risks of As, Pb and Zn were slightly higher than the national average [31]. In general, the daily average exposure dose and non-carcinogenic risk for different people varied slightly, showing that children were higher than adults were. The average hazard quotients of PHEs decreased in the order of *HQ_Mn_* > *HQ_Cr_* > *HQ_As_* > *HQ_Pb_* > *HQ_Co_* > *HQ_Ni_* > *HQ_Cu_* > *HQ_Zn_*, and the contribution rates to *HI* were 48.87%–73.30%, 13.65%–25.48%, 7.52%–16.09%, 1.29%–1.67%, 0.70%–1.49%, 0.13%–0.28%, 0.10%–0.21% and 0.15%–0.16%, respectively. For all PHEs, the average daily exposure dose of ingestion was almost 10^3^ times that of inhalation and dermal contact. Similarly, the non-carcinogenic risk of most PHEs through ingestion obviously exceeded the other pathways, and risk caused by dermal contact of Mn also accounted for a large proportion. Therefore, the non-carcinogenic risk was mainly contributed by the exposure pathway of ingestion in Cr and dermal contact in Mn. Although there was no non-carcinogenic risk in the study area, the HQ of children were nearly three times that of adults, indicating that children were more vulnerable to PHEs (Figure 8).

#### 3.4.2. Carcinogenic Risk Assessment from As, Co, Ni, Gr and Pb

Due to only As, Co, Ni, Cr, and Pb have carcinogenic slope factor parameters, the *ADD_ing_*, *RI_i,_* and *RI* of adults and children through three exposure routes (oral intake, respiratory inhalation, and dermal contact) were evaluated. As shown in Table 5, the daily average exposure of children is higher than that of adults under different exposure routes, and the *RI_i_* and *RI* of children are higher than that of adults. The average *RI* of PHEs is following the order of *RI_Cr_* > *RI_As_* > *RI_Pb_* > *RI_Co_* > *RI_Ni_*, with the contribution rate of 83.04%–83.05%, 16.49%–16.52%, 0.42%–0.43%, 0.01%–0.04%, 0.0016%–0.008%, respectively. This indicated that the *RI* of PHEs (As, Co, Ni, Cr, and Pb) in the soil of the study area is at an acceptable risk level, which is mainly contributed by the oral exposure of Cr and As. From different exposure routes, the daily average exposure of oral intake is 2–3 orders of magnitude higher than that of inhalation and dermal contact. The *RI_ing_* of As, Cr, and Pb in oral intake was much higher than that of *RI_inh_* and *RI_derm_*.

## 4. Discussion

### 4.1. The Influence of Different Land Use on PHEs Enrichment in Coastal Tideland Region

High-intensity reclamations have resulted in drastic changes in land use of tidal flats, which have made ecosystems more fragile and sensitive, including the increased risk of soil pollution [19]. Table 3 indicated that the concentrations of PHEs in surface and subsurface soil layers were almost equal, but the maximum and minimum concentrations of whole sites differed greatly. Furthermore, soil depth was observed to have less influence on the concentration of PHEs than land use. Soil environmental status of the study area was generally in good condition due to the average concentrations of all PHEs lower than the environmental quality standard for soils in China (Table 3). However, the maximum concentration of some PHEs was close to or exceeded the limits of first grade (As and Pb) and second grade (Cr) of the environmental quality standard, which might be affected by reclamation activities, specifically manifested in the changes of land-use intensity and soil properties [24,26,45]. The traditional evolution pathway of land use in the reclaimed coastal areas in eastern China was mudflat→halophyte land→aquaculture land→arable land→construction land [51], which showed that the land-use intensity gradually increased with the increase of human activity interference. In this study, the concentrations of most PHEs (except Co) were in the following order: aquaculture land > industrial land/cropland > forestland/residential land > vegetable land > tideland/halophyte land (Figure 3). Yao et al. [52] also reported similar conclusions after soil investigation on the reclaimed tideland of Dongtai City, Jiangsu Province, which found there was no significant excess of soil PHEs, while the total ecological risk and polluted level showed the trend following the order: industrial park > residential area > suburb farmland > newly-reclaimed tidal flats. ANOVA (Figure 3) illustrated that the concentrations of As, Cu, Pb and Zn in soil after 30 years of reclamation (industrial land, aquaculture land, farmland, residential land, and vegetable land) were significantly higher than those in uncultivated coastal wetlands (tideland and halophyte land), which might be caused by changes in soil physical and chemical properties under reclamation activities [19]. It is reported that the sand content, salinity, and pH gradually decreased, while the silt and clay content and organic matter content gradually increased following tidal land reclamation, which finally reached a stable state after 30 years reclamation [14,19]. The dynamics of soil physicochemical properties have been proven as the key factors affecting the mobility of PHEs in soil [14]. Specifically, fine-grained sediments are easy to solidify PHEs in soil [53], the increase of SOM promotes PHEs accumulation [1,24], and the soluble PHEs in pore water decreases with the decreasing of soil salinity after reclamation [27].

The sources of PHEs in the study area were complex based on the results of enrichment analysis, correlation analysis, and cluster analysis. The *EFs* were observed less than 2.0 in the study area, which indicated the sources of soil PHEs were largely contributed by parent materials and partly determined by human activities, such as land use. The enrichment analysis showed Co (0.86–1.39), Cr (0.96–2.01), Mn (1.21–1.75), Ni (1.11–1.84), Pb (0.78–1.85), and Zn (0.97–1.61) were generally in slight enrichment, while Cr, Ni, and Pb in some sampling sites were close to moderate enrichment (Figure 4). Therefore, it is necessary to pay attention to the pollution of Cr, Ni, and Pb in the future reclamation and utilization of tidal land. The EFs of the determined PHEs in halophyte land, tideland, and forestland were generally classified as low concentrations group, and that in cropland and aquaculture land were classified as high concentrations group. Similarly, Sowan et al. [26] reported that the PHEs contamination in municipality areas, industrial zones, and dockyard were more serious, while shrimp farming and traditional land uses such as salt marshes, paddy fields, orchards, and mangrove forests showed low levels of metals in Pattani Bay. These conclusions proved the intensity of human activity further influenced the soil PHEs enrichment [7,54].

It could be seen in Table 3, the highest concentration of Cu (14.10 mg·kg^−1^) was much smaller than the background of Jiangsu (22.3 mg·kg^−1^), and the EFs of Cu did not exceed 1.0 in all land-use types. This indicates that no significant Cu enrichment was observed in the study area. Actually, the average concentration of Cu (8.10 mg·kg^−1^) was close to the deep sediment of coastal mudflat in Jiangsu (13.0 mg·kg^−1^) [49], which indicated that Cu most likely originated in natural factors and was rarely disturbed by anthropogenic activities [55].

Co and Mn might have the same sources and be slightly accumulated by the influence of human activities, as their correlation coefficient was 0.58 and the average EF ranged from 1 to 1.5. Table 3 showed that the average concentrations of Co and Mn were less than the background parameters, and their concentrations in the surface (8.00 mg·kg^−1^, 457.49 mg·kg^−1^) were lower than that in the subsurface layer (8.01 mg·kg^−1^, 460.03 mg·kg^−1^). This phenomenon was primarily attributed to the natural leaching, which indicated that Co and Mn were controlled by natural geological processes [15]. Li et al. [7] and Franco-Uría et al. [56] also presented that Co and Mn were originated from a lithogenic source [24]. The EF of Co and Mn in tideland (EF = 1.14, 1.43) and halophyte land (EF = 1.16, 1.39) were observed to be slightly lower than that in aquaculture land (EF = 1.17, 1.47), which also revealed that the Co and Mn background content in the tidal flats under the natural sedimentary process was accounted for high proportions [24]. Co and Mn highly accumulated in aquaculture land compared with other land-use types. The marine aquaculture fish feed usually contains Zn, Cu, Cd, Fe, Mn, Co, Ni, P [55], and it may be a major source of Co and Mn. In addition, the water submerged environment promotes metals and S^2-^ associate and precipitate to insoluble metal sulfides under redox status [57]. Thereby, natural sources mostly contribute to the distribution of Co and Mn, and aquaculture is another source. 

Cr seemed to be an isolated element, having low correlations with other elements (r < 0.4) [58]. Even if Cr originates from a natural source which has been extensively mentioned in many pieces of literature [15,59]. In this study, the distribution of Cr was mainly affected by agricultural input as the average EF of Cr in cropland (EF = 1.36) and aquaculture land (EF = 1.30) was significantly higher than that of others. The average concentration of determined Cr (52.50 mg·kg^−1^) in cropland was higher than that in phosphate fertilizers (47.3 mg·kg^−1^), whereas much less than that in sludge (86.73 mg·kg^−1^) [24]. Consequently, the enrichment of Cr in the study area may be the result of the application of fertilizer using sludge as the raw material. Field investigation found printing and dyeing mills and mining enterprises using Cr compounds around the reclamation area [60]. In summary, sewage irrigation and fish feed fish have become important sources of soil Cr in this area [55,61].

The slight accumulation of soil As appeared locally rather than the whole area (*EF_average_* = 0.58, *EF_max_* = 1.19). As, Cu and Pb had homology as the correlation coefficients among them all exceeded 0.5. It had been concluded that Cu originated from natural factors, which indirectly revealed that As and Pb were partially derived from natural parent materials. The average concentration and EF of As in industrial land were significantly higher than those in other land use types, indicating that the industrial activities might be another source of As. Huang et al. [62] and Khairy et al. [63] reported that As was related to the emissions of industrial flue gas, sewage, and sludge. The average EF of Pb ranged from 0.93 to 1.21 under different land-use types and tended to accumulate in aquaculture land, industrial land, vegetable land, and cropland. The cropland and fishponds were adjacent to each other, which might cause mutual pollution. It is widely reported that pesticides and fertilizers (e.g., phosphate fertilizers, nitrate fertilizers, and organic fertilizers) applied in agricultural soils caused high concentrations of Cd, Pb, and Zn [15,64].

Zn-Ni-Cu were significantly correlated (r > 0.75), which revealed that partly, sources of Zn and Ni might derive from natural parent materials as well as Cu. This was consistent with the result of Sun et al. [59]. Slight enrichment of Zn and Ni occurred in aquaculture land, industrial land, and cropland (1.30 < EF < 1.60, Figure 4). Zn accumulated in fishery sediments may be attributed to the input of fish feed or fish manure [55]. In addition, the application of poultry manure in agricultural land increased Zn accumulation [15,56]. Furthermore, the emission of automobile exhaust and wear of the tires significantly affected the Zn and Ni concentrations in road dust, and then entered the surrounding soil through atmospheric sedimentation [15,63]. Ni might be directly related to the “three wastes” (including some domestic waste) emissions during the production of batteries near the study area as the battery slag and wastewater contains high concentrations of Pb, Cd, Ni, etc. [49].

### 4.2. The PHEs Health Risk in Coastal Tideland Reclamation Area

It is found that the total non-carcinogenic risk of soil PHEs in the study area is at the no non-carcinogenic risk level, while the total carcinogenic risk is at the acceptable risk level. This is consistent with the result that the average concentration of PHEs in Table 3, indicating that the concentration of soil PHEs in the study area has no obvious harm to human health. It is worth noting that the carcinogenic risk index of Cr (1.50 × 10^−5^–2.30 × 10^−5^) and As (2.99 × 10^−6^–4.58 × 10^−6^) exceeds the maximum acceptable carcinogenic risk of the human body. This might be attributed to the high slope coefficient of the carcinogenic risk of Cr and As [31,43]. It is reported that As is a carcinogen for lung cancer and skin cancer, and excessive intake of Cr will cause respiratory and digestive diseases [3]. Accordingly, As and Cr should receive more attention in future soil environmental monitoring of coastal reclamation areas. Tideland reclamation accelerated the transformation from bare flat to cultivated land, aquaculture land and construction land, which had an important impact on the health risk of PHEs in soil. In this study, the highest non-carcinogenic risk and the carcinogenic risk appeared in industrial land, followed by farmland and vegetable land (Figure 8). Yang et.al [60] found that the health risks of industrial areas were higher than that of agricultural areas in China. Table 3 and Table 4 showed that the ratio of average daily exposure dose between ingestion and inhalation, dermal contact was all close to 10^3^. However, the non-carcinogenic risk was mainly contributed by ingestion and dermal contact of Mn and ingestion of Cr (Table 4). Carcinogenic risk assessment (Table 5) showed that ingestion was the main exposure route for As, Cr, and Pb, while the inhalation was the unique exposure pathway for Co and Ni. This might be due to the increased risk exposure pathway by the reclamation of tideland. The smoke discharged by industry and transportation enters the human body through inhalation, while the rest enters the soil through natural sedimentation and rain. PHEs from industrial activities and agricultural fertilization management accumulate continuously after entering the soil and migrate in different systems (such as water systems and crops), thereby entering the human body through the food chain, resulting in harm to human health [3,65]. Besides, compared with daily average exposure, non-carcinogenic risk index and carcinogenic risk index, it can be found that children are more vulnerable to PHEs in soil than adults (Figure 8), which is similar to previous research results [44,60,65]. It is mainly because the children’s immune resistance is much lower than that of adults, which is more sensitive to environmental pollution. In addition, children’s exposure to soil due to outdoor activities is higher than that of adults, and children’s physical behavior (such as finger sucking) makes their health risk higher than adults [3,65].

## 5. Conclusions

The enrichment, sources apportionment, and health risk assessment of eight PHEs (As, Co, Cr, Cu, Mn, Ni, Pb, and Zn) were identified in Jiangsu coastal reclamation area, eastern China. In general, none of the PHEs we concerned about exceeding the environmental quality standard for soils in China. Enrichment analysis showed that As and Cu were not enriched, while other elements were slightly enriched. Land use is observed to have an obvious enrichment effect on most of the PHEs, which resulted in a relatively high level of PHEs enrichment in aquaculture land, industrial land, and cropland compared with other land use types, such as tideland and halophyte land. The correlation analysis and hierarchically cluster divided PHEs into five categories: (1) Cu; (2) Co and Mn; (3) Cr; (4) As and Pb; (5) Zn and Ni. Additionally, it was observed that the Cu was completely derived from natural parent materials; the Mn and Co were mainly controlled by natural sources and aquaculture is another source, and; the Cr was an independent element and was mainly derived from agricultural fertilization and aquaculture activities. However, the As, Pb, Ni, and Zn are governed by both weathering of parent rock and human activities, including agricultural activities, industrial production, and transportation emissions. The health risk assessment showed that the soil PHEs potentially had no non-carcinogenic risk to the public, while there was an acceptable probability to have cancer due to Cr and As. Meanwhile, children were more susceptible to harm of soil PHEs than that of adults. Although the soil PHEs in the study area had not caused significant negative effects on the environment and individuals, the uncertainty of some PHEs (Pb, As, and Cr) was still high. That is to say, it may increase PHEs pollution and further increase the probability of human body damage if land management could not be rationally planned in the future.

## Figures and Tables

**Table 1:** Parameters used to evaluate exposure risks of soil metals.

Parameters	Definitions	Values		References
		Adult	Children	
*C_Soil_*	Concentration of potentially harmful elements in soil/(mg·kg^−1^)	Average		
*CF*	Conversion coefficient	1 × 10^−6^ kg·mg^−1^		[38]
*IngR*	Ingestion rate of soil/(mg·d^−1^)	100	200	[38]
*EF*	Exposure frequency/(d·a^−1^)	365		[39]
*ED*	Exposure duration/year	24	6	[38]
*BW*	Weight/kg	60.6	19.6	[40]
*AT_nc_*	Averaged time of carcinogenic impact/day	70×365		[38]
*AT_ca_*	Averaged time of non-carcinogenic impact/day	ED×365		[38]
*InhR*	inhalation rate of soil/(d·a^−1^)	20	7.65	[38]
*PEF*	Emission factor/(m^3^·kg^−1^)	1.36×10^9^		[38]
*SA*	Exposed skin surface area/cm^2^	5 700	2 800	[40]
*SL*	Adherence factor/(mg·cm^−2^·d^−1^)	0.07	0.2	[38]
*ABS*	Dermal absorption factor	0.001		[38]

**Table 2:** Reference dose and slope factor of potentially harmful elements (PHEs) under different exposure pathways.

Parameter		As	Co	Cr	Cu	Mn	Ni	Pb	Zn
Ingestion	*RfD*/(mg·kg^−1^·d^−1^)	3.00 × 10^−4^	2.00 × 10^−2^	3.00 × 10^−3^	4.00 × 10^−2^	4.60 × 10^−2^	2.00 × 10^−2^	3.50 × 10^−3^	3.00 × 10^−1^
	*SF*/(mg·kg^−1^·d^−1^)	1.50	—	5.00 × 10^−1^	—	—	—	8.50 × 10^−3^	—
Inhalation	*RfD*/(mg·kg^−1^·d^−1^)	3.00 × 10^−4^	5.71 × 10^−6^	2.86 × 10^−5^	4.00 × 10^−02^	1.43 × 10^−5^	2.06 × 10^−2^	3.52 × 10^−3^	3.00 × 10^−1^
	*SF*/(mg·kg^−1^·d^−1^)	1.51 × 10 ^1^	9.80	4.20 × 10^1^	—	—	8.40 × 10^−1^	—	—
D × 10rmal Contact	*RfD*/(mg·kg^−1^·d^−1^)	1.23 × 10 ^−4^	1.60 × 10^−5^	6.00 × 10^−5^	1.20 × 10^−2^	1.84 × 10^−5^	5.40 × 10^−3^	5.25 × 10^−3^	6.00 × 10^−2^
	*SF*/(mg·kg^−1^·d^−1^)	3.66	—	—	—	—	—	—	—

PHEs with carcinogenic risk are As, Cd, Cr, Ni and Pb; others are non-carcinogenic metals. “—” in the table means not available.

**Table 3:** Statistical description of PHEs contents in the study area (mg·kg^−1^).

PHEs	Min	Max	Mean	Mean_1_	Mean_2_	S.D	S.D_1_	S.D_2_	CV	Background of Jiangsu [32]	Background of China [48]	Risk Control Standard China (1)/(2)/(3)
As	0.44	11.08	3.31	3.29	3.33	2.09	1.41	1.62	60.06	10.0	15.0	15/25/30
Co	5.04	10.81	8.00	8.00	8.01	1.13	1.15	1.04	14.09	12.6	−	−
Cr	38.94	116.53	52.50	51.84	53.16	11.60	7.42	14.52	22.10	77.8	90.0	90/200/300
Cu	4.13	14.10	8.10	8.36	7.85	2.21	2.35	1.97	27.22	22.3	35.0	30/100/400
Mn	374.19	643.80	458.76	457.49	460.03	35.32	39.10	30.71	7.70	585	−	−
Ni	16.49	28.25	21.47	21.35	21.58	2.09	2.31	1.81	9.73	26.7	40.0	40/50/200
Pb	9.18	38.76	15.90	16.31	15.49	3.52	3.39	3.57	22.14	26.2	35.0	35/300/500
Zn	33.37	62.04	45.45	46.82	44.07	5.76	6.22	4.84	12.67	62.6	100.0	100/250/500

*Risk control standard China* is Environmental quality standard for soils (GB 15618-1995) and has three levels of standards. (1) means the limit value of the soil environmental quality to protect the regional natural ecology and maintain the natural background. (2) means the limit value of the soil to ensure agricultural production and maintain human health; (3) means the limit value of the soil to ensure the agricultural and forestry production and normal plant growth. Mean_1_ and Mean_2_ represent the average concentration of PHEs in surface and subsurface. S.D_1_ and S.D_2_ represent standard deviations for the surface and subsurface concentrations.

**Table 4:** Daily exposure and non-carcinogenic risk index for adults and children with PHEs and pathways in 0–40 cm soil layer.

Metals	Age Group	*ADD_ing_*	*ADD_inh_*	*ADD_derm_*	*HQ_ing_*	*HQ_inh_*	*HQ_derm_*	*HQ_i_*
As	Adult	5.74 × 10^−6^	8.44 × 10^−10^	2.29 × 10^−8^	1.91 × 10^−2^	2.81 × 10^−6^	1.86 × 10^−4^	1.93 × 10^−2^
	Childen	3.55 × 10^−5^	9.99 × 10^−10^	3.48 × 10^−8^	1.18 × 10^−1^	3.33 × 10^−6^	2.83 × 10^−4^	1.19 × 10^−1^
Co	Adult	1.32 × 10^−5^	1.95 × 10^−9^	5.28 × 10^−8^	6.62 × 10^−4^	3.41 × 10^−4^	3.30 × 10^−3^	4.30 × 10^−3^
	Children	8.18 × 10^−5^	2.30 × 10^−9^	8.02 × 10^−8^	4.09 × 10^−3^	4.03 × 10^−4^	5.01 × 10^−3^	9.51 × 10^−3^
Cr	Adult	8.66 × 10^−5^	1.27 × 10^−8^	3.46 × 10^−7^	2.89 × 10^−2^	4.45 × 10^−4^	5.76 × 10^−3^	3.51 × 10^−2^
	Children	5.36 × 10^−4^	1.51 × 10^−8^	5.25 × 10^−7^	1.79 × 10^−1^	5.27 × 10^−4^	8.75 × 10^−3^	1.88 × 10^−1^
Cu	Adult	1.34 × 10^−5^	1.97 × 10^−9^	5.35 × 10^−8^	3.35 × 10^−4^	4.93 × 10^−8^	4.46 × 10^−6^	3.39 × 10^−4^
	Children	8.29 × 10^−5^	2.33 × 10^−9^	8.12 × 10^−8^	2.07 × 10^−3^	5.83 × 10^−8^	6.77 × 10^−6^	2.08 × 10^−3^
Mn	Adult	7.57 × 10^−4^	1.11 × 10^−7^	3.02 × 10^−6^	1.65 × 10^−2^	7.79 × 10^−3^	1.64 × 10^−1^	1.88 × 10^−1^
	Children	4.68 × 10^−3^	1.32 × 10^−7^	4.59 × 10^−6^	1.02 × 10^−1^	9.21 × 10^−3^	2.49 × 10^−1^	3.60 × 10^−1^
Ni	Adult	3.54 × 10^−5^	2.53 × 10^−7^	1.41 × 10^−7^	1.77 × 10^−3^	2.53 × 10^−7^	2.62 × 10^−5^	1.80 × 10^−3^
	Children	2.19 × 10^−4^	2.99 × 10^−7^	2.15 × 10^−7^	1.10 × 10^−2^	2.99 × 10^−7^	3.98 × 10^−5^	1.10 × 10^−2^
Pb	Adult	2.62 × 10^−5^	3.86 × 10^−9^	1.05 × 10^−7^	7.50 × 10^−3^	1.10 × 10^−6^	1.99 × 10^−5^	7.52 × 10^−3^
	Children	1.62 × 10^−4^	4.56 × 10^−9^	1.59 × 10^−7^	4.64 × 10^−2^	1.30 × 10^−6^	3.03 × 10^−5^	4.64 × 10^−2^
Zn	Adult	7.50 × 10^−5^	1.10 × 10^−8^	2.99 × 10^−7^	2.50 × 10^−4^	3.68 × 10^−8^	4.99 × 10^−6^	2.55 × 10^−4^
	Children	4.64 × 10^−4^	1.30 × 10^−8^	4.55 × 10^−7^	1.55 × 10^−3^	4.35 × 10^−8^	7.58 × 10^−6^	1.55 × 10^−3^
*HI*	Adult	— ^1^	—	—	—	—	—	2.57 × 10^−1^
	Children	—	—	—	—	—	—	7.37 × 10^−1^

^1^ “—” in the table means not available.

**Table 5:** Daily exposure and carcinogenic risk index for adults and children with PHEs and pathways in 0–40 cm soil layer.

Metals	Age Group	*ADD_ing_*	*ADD_inh_*	*ADD_derm_*	*CR_ing_*	*CR_inh_*	*CR_derm_*	*CR_i_*
As	Adult	1.97 × 10^−6^	2.90× 10^−10^	7.86 × 10^−9^	2.95 × 10^−6^	4.37 × 10^−9^	2.88 × 10^−8^	2.99 × 10^−6^
	Children	3.04 × 10^−6^	8.56 × 10^−11^	2.98 × 10^−9^	4.57 × 10^−6^	1.29 × 10^−9^	1.09 × 10^−8^	4.58 × 10^−6^
Co	Adult	4.54 × 10^−6^	6.67 × 10^−10^	1.81 × 10^−8^	— ^1^	6.54 × 10^−9^	—	6.54 × 10^−9^
	Children	7.01 × 10^−6^	1.97 × 10^−10^	6.87 × 10^−9^	—	1.93 × 10^−9^	—	1.93 × 10^−9^
Cr	Adult	2.97 × 10^−5^	4.37 × 10^−9^	1.19 × 10^−7^	1.49 × 10^−5^	1.83 × 10^−7^	—	1.50 × 10^−5^
	Children	4.59 × 10^−5^	1.29 × 10^−9^	4.50 × 10^−8^	2.30 × 10^−5^	5.42 × 10^−8^	—	2.30 × 10^−5^
Ni	Adult	1.21 × 10^−5^	1.79 × 10^−9^	4.85 × 10^−8^	—	1.50 × 10^−9^	—	1.50 × 10^−9^
	Children	1.88 × 10^−5^	5.28 × 10^−10^	1.84 × 10^−8^	—	4.44 × 10^−10^	—	4.44 × 10^−10^
Pb	Adult	9.00 × 10^−6^	1.32 × 10^−9^	3.59 × 10^−8^	7.65 × 10^−8^	—	—	7.65 × 10^−8^
	Children	1.39 × 10^−5^	3.91 × 10^−10^	1.36 × 10^−8^	1.18 × 10^−7^	—	—	1.18 × 10^−7^
*CR*	Adult	—	—	—	—	—	—	1.81 × 10^−5^
	Children	—	—	—	—	—	—	2.77 × 10^−5^

^1^ “—” in the table means not available.

## References

[1] Qian Z., Guilin H., Man L., Xiaoqiang L., Lingqing W., Bin L. (2019). Distribution and Contamination Assessment of Soil Heavy Metals in the Jiulongjiang River Catchment, Southeast China. Int. J. Environ. Res. Public Health.

[2] Cheng S. (2003). Heavy metal pollution in China: Origin, pattern and control. Environ. Sci. Pollut. Res..

[3] Zhao H., Xia B., Fan C., Zhao P., Shen S. (2012). Human health risk from soil heavy metal contamination under different land uses near Dabaoshan Mine, Southern China. Sci. Total Environ..

[4] Lee J.-S., Chon H.-T., Kim K.-W. (1998). Migration and dispersion of trace elements in the rock–soil–plant system in areas underlain by black shales and slates of the Okchon Zone, Korea. J. Geochem. Explor..

[5] Wei B., Yang L. (2010). A review of heavy metal contaminations in urban soils, urban road dusts and agricultural soils from China. Microchem. J..

[6] Li J., Pu L., Zhu M., Liao Q., Wang H., Cai F. (2014). Spatial pattern of heavy metal concentration in the soil of rapid urbanization area: A case of Ehu Town, Wuxi City, Eastern China. Environ. Earth Sci..

[7] Li J., Pu L., Liao Q., Zhu M., Dai X., Xu Y., Zhang L., Hua M., Jin Y. (2015). How anthropogenic activities affect soil heavy metal concentration on a broad scale: A geochemistry survey in Yangtze River Delta, Eastern China. Environ. Earth Sci..

[8] Li Z., Ma Z., Kuijp T.J.V.D., Yuan Z., Huang L. (2013). A Review of Soil Heavy Metal Pollution From Mines in China: Pollution and Health Risk Assessment. Sci. Total Environ..

[9] Krishna A.K., Govil P.K. (2004). Heavy metal contamination of soil around Pali Industrial Area, Rajasthan, India. Environ. Geol..

[10] Lv J., Liu Y., Zhang Z., Dai J. (2013). Factorial kriging and stepwise regression approach to identify environmental factors influencing spatial multi-scale variability of heavy metals in soils. J. Hazard. Mater..

[11] Ferri R., Donna F., Smith D.R., Guazzetti S., Zacco A., Rizzo L., Bontempi E., Zimmerman N.J., Lucchini R.G. (2012). Heavy metals in soil and salad in the proximity of historical ferroalloy emission. J. Environ. Protect..

[12] Zhang X., Zhong T., Liu L., Ouyang X. (2015). Impact of soil heavy metal pollution on food safety in China. PLoS ONE.

[13] Carrero J.A., Arrizabalaga I., Bustamante J., Goienaga N., Arana G., Madariaga J.M. (2013). Diagnosing the traffic impact on roadside soils through a multianalytical data analysis of the concentration profiles of traffic-related elements. Sci. Total Environ..

[14] Zheng R., Jiale Z., Xiu Z., Chao M., Li W., Xiaojiang G. (2016). Land use effects on the distribution and speciation of heavy metals and arsenic in coastal soils on Chongming Island in the Yangtze River Estuary, China. Pedosphere.

[15] Lv J., Liu Y., Zhang Z., Dai J., Dai B., Zhu Y. (2015). Identifying the origins and spatial distributions of heavy metals in soils of Ju country (Eastern China) using multivariate and geostatistical approach. J. Soil. Sediment..

[16] Tang L., Deng S., Tan D., Long J., Lei M. (2019). Heavy metal distribution, translocation, and human health risk assessment in the soil-rice system around Dongting Lake area, China. Environ. Sci. Pollut. Res..

[17] Gedan K.B., Silliman B.R., Bertness M.D. (2009). Centuries of Human-Driven Change in Salt Marsh Ecosystems. Annu. Rev. Mar. Sci..

[18] Crossland C.J., Baird D., Ducrotoy J.-P., Lindeboom H., Buddemeier R.W., Dennison W.C., Maxwell B.A., Smith S.V., Swaney D.P., Crossland C.J., Kremer H.H., Lindeboom H.J., Marshall Crossland J.I., Le Tissier M.D.A. (2005). The Coastal Zone—A Domain of Global Interactions. Coastal Fluxes in the Anthropocene: The Land-Ocean Interactions in the Coastal Zone Project of the International Geosphere-Biosphere Programme.

[19] Xu C.Y., Pu L.J., Zhu M. (2018). Effect of reclamation activity on coastal ecological environment: Progress and perspectives. Acta Ecol. Sin..

[20] Wang F., Wall G. (2010). Mudflat development in Jiangsu Province, China: Practices and experiences. Ocean Coast. Manage..

[21] Wang L., Coles N.A., Wu C., Wu J. (2014). Spatial variability of heavy metals in the coastal soils under long-term reclamation. Estua. Coast. Shelf Sci..

[22] Miao X., Miao D., Hao Y., Xie Z., Zou S. (2019). Potential health risks associated to heavy metal contamination of soils in the Yellow River Delta, China. J. Coast. Conserv..

[23] Machender G., Dhakate R., Prasanna L., Govil P. (2011). Assessment of heavy metal contamination in soils around Balanagar industrial area, Hyderabad, India. Environ. Earth Sci..

[24] Bai J., Xiao R., Cui B., Zhang K., Wang Q., Liu X., Gao H., Huang L. (2011). Assessment of heavy metal pollution in wetland soils from the young and old reclaimed regions in the Pearl River Estuary, South China. Environ. Pollut..

[25] Mao L., Liu L., Yan N., Li F., Tao H., Ye H., Wen H. (2020). Factors controlling the accumulation and ecological risk of trace metal (loid) s in river sediments in agricultural field. Chemosphere.

[26] Sowana A., Shrestha R.P., Parkpian P., Pongquan S. (2011). Influence of Coastal Land Use on Soil Heavy-Metal Contamination in Pattani Bay, Thailand. J. Coast. Res..

[27] Zhao J., Gao X., Yang J. (2017). Influences of hydrological regime on heavy metal and salt ion concentrations in intertidal sediment from Chongming Dongtan, Changjiang River estuary, China. Chin. J. Oceanol. Limn..

[28] Xie X.F., Pu L.J., Zhu M., Xu Y., Wang X.H. (2019). Linkage between soil salinization indicators and physicochemical properties in a long-term intensive agricultural coastal reclamation area, Eastern China. J. Soil. Sediment..

[29] Xie X.F., Pu L.J., Wang Q.Q., Zhu M., Xu Y., Zhang M. (2017). Response of soil physicochemical properties and enzyme activities to long-term reclamation of coastal saline soil, Eastern China. Sci. Total Environ..

[30] Xia F., Hu B., Shao S., Xu D., Zhou Y., Zhou Y., Huang M., Li Y., Chen S., Shi Z. (2019). Improvement of Spatial Modeling of Cr, Pb, Cd, As and Ni in Soil Based on Portable X-ray Fluorescence (PXRF) and Geostatistics: A Case Study in East China. Int. J. Environ. Res. Public Health.

[31] Chen H., Teng Y., Lu S., Wang Y., Wang J. (2015). Contamination features and health risk of soil heavy metals in China. Sci. Total Environ..

[32] Liao Q.L., Liu C., Xu Y., Jin Y., Wu J.Z., Hua M., Zhu B.W., Weng Z.H. (2011). Geochemical baseline values of elements in soil of Jiangsu Province. Geol. China.

[33] Lu X., Zhang X., Li L.Y., Chen H. (2014). Assessment of metals pollution and health risk in dust from nursery schools in Xi’an, China. Environ. Res..

[34] Doležalová W.H., Mihočová S., Chovanec P., Pavlovský J. (2019). Potential Ecological Risk and Human Health Risk Assessment of Heavy Metal Pollution in Industrial Affected Soils by Coal Mining and Metallurgy in Ostrava, Czech Republic. Int. J. Environ. Res. Public Health.

[35] USEPA (1998). Risk Assessment Guidance for Superfund (RAGS).

[36] Wu S., Peng S., Zhang X., Wu D., Luo W., Zhang T., Zhou S., Yang G., Wan H., Wu L. (2015). Levels and health risk assessments of heavy metals in urban soils in Dongguan, China. J. Geochem. Explor..

[37] USEPA (1989). Risk assessment guidance for superfund. Saúde Pública.

[38] USEPA (2013). Electronic Code of Federal Regulations, Title 40—Protection of Environment. Part 423d Steam Electric Power Generating Point Source Category.

[39] Yuswir N.S., Praveena S.M., Aris A.Z., Ismail S.N.S., Hashim Z. (2015). Health Risk Assessment of Heavy Metal in Urban Surface Soil (Klang District, Malaysia). Bull. Environ. Contam. Toxic..

[40] Ministry of Environmental Protection, China (2013). Exposure Factors Handbook of Chinese Population (Adults, Children).

[41] Yin Y.M., Zhao W.T., Huang T., Cheng S.G., Zhao L.Z., Yu C.C. (2018). Distribution characteristics and health risk assessment of heavy metals in a soil-rice system in an e-waste dismantling area. Environ. Sci..

[42] USEPA (2009). National Center for Environmental Assessment. Highlights of the Child-Specific Exposure Factors Handbook (Final Report).

[43] Ferreira-Baptista L., De Miguel E. (2005). Geochemistry and risk assessment of street dust in Luanda, Angola: A tropical urban environment. Atmos. Environ..

[44] Ihedioha J., Ukoha P., Ekere N. (2017). Ecological and human health risk assessment of heavy metal contamination in soil of a municipal solid waste dump in Uyo, Nigeria. Environ. Geochem. Health.

[45] Ma C., Zheng R., Zhao J., Han X., Wang L., Gao X., Zhang C. (2015). Relationships between heavy metal concentrations in soils and reclamation history in the reclaimed coastal area of Chongming Dongtan of the Yangtze River Estuary, China. J. Soil. Sediment..

[46] Xu L., Yang W., Jiang F., Qiao Y., Yan Y., An S., Leng X. (2018). Effects of reclamation on heavy metal pollution in a coastal wetland reserve. J. Coast. Conserv..

[47] Lv J.S., He H.C. (2018). Identifying the Origins and Spatial Distributions of Heavy Metals in the Soils of the Jiangsu Coast. Environ. Sci..

[48] Xia J.C., Cai Z., Wang H., Wu M., Liang W. (1995). Environmental Quality Standard for Soils (GB15618-1995).

[49] Liao Q.L., Ren J.H., Jiang L., Zhang X., Zhu B.W., Li W.B., Wang Z.Y. (2018). Distribution of heavy metal elements in fluvial sediments of typical areas in Jiangsu Province and their pollution sources. J. Geol..

[50] Abdallah M.A.M. (2011). Ecological risk assessment of heavy metals from the surficial sediments of a shallow coastal lagoon, Egypt. Environ. Technol..

[51] Zhao S.S., Liu Y.X., Li M.C., Sun C., Zhou M.X., Zhang H.X. (2015). Analysis of Jiangsu tidal flats reclamation from 1974 to 2012 using remote sensing. China Ocean Eng..

[52] Yao R.J., Yang J.S., Xie W., Wu D., Bai Y., Yu S., Zhang X. (2016). Contents and spatio-temporal variability of soil heavy metals in the coastal mud-flat area of north Jiangsu Province. China Environ. Sci..

[53] Zhang W., Yu L., Hutchinson S.M., Xu S., Gao X. (2001). China’s Yangtze Estuary: I. Geomorphic influence on heavy metal accumulation in intertidal sediments. Geomorphology.

[54] Li H.B., Yu S., Li G.-L., Liu Y., Yu G.-B., Deng H., Wu S.-C., Wong M.-H. (2012). Urbanization increased metal levels in lake surface sediment and catchment topsoil of waterscape parks. Sci. Total Environ..

[55] Dean R.J., Shimmield T.M., Black K.D. (2007). Copper, zinc and cadmium in marine cage fish farm sediments: An extensive survey. Environ. Pollut..

[56] Franco-Uría A., López-Mateo C., Roca E., Fernández-Marcos M.L. (2009). Source identification of heavy metals in pastureland by multivariate analysis in NW Spain. J. Hazard. Mater..

[57] Laing G.D., Rinklebe J., Vandecasteele B., Meers E., Tack F.M.G. (2009). Trace metal behaviour in estuarine and riverine floodplain soils and sediments: A review. Sci. Total Environ..

[58] Peris M., Recatalá L., Micó C., Sánchez R., Sánchez J. (2008). Increasing the Knowledge of Heavy Metal Contents and Sources in Agricultural Soils of the European Mediterranean Region. Water Air Soil Pollut..

[59] Sun C., Liu J., Wang Y., Sun L., Yu H. (2013). Multivariate and geostatistical analyses of the spatial distribution and sources of heavy metals in agricultural soil in Dehui, Northeast China. Chemosphere.

[60] Yang Q., Li Z., Lu X., Duan Q., Huang L., Bi J. (2018). A review of soil heavy metal pollution from industrial and agricultural regions in China: Pollution and risk assessment. Sci. Total Environ..

[61] Zhang W., Liu X., Cheng H., Zeng E.Y., Hu Y. (2012). Heavy metal pollution in sediments of a typical mariculture zone in South China. Mar. Pollut. Bull..

[62] Huang H., Lin C., Yu R., Yan Y., Li H. (2019). Contamination assessment, source apportionment and health risk assessment of heavy metals in paddy soils of Jiulong River Basin, Southeast China. RSC Adv..

[63] Khairy M.A., Barakat A.O., Mostafa A.R., Wade T.L. (2011). Multielement determination by flame atomic absorption of road dust samples in Delta Region, Egypt. Microchem. J..

[64] Bai J., Cui B., Yang Z., Xu X., Ding Q., Gao H. (2010). Heavy metal contamination of cultivated wetland soils along a typical plateau lake from southwest China. Environ. Earth Sci..

[65] Wang Y., Qiao M., Liu Y., Zhu Y. (2012). Health risk assessment of heavy metals in soils and vegetables from wastewater irrigated area, Beijing-Tianjin city cluster, China. J. Environ. Sci..

